# Supplementary material for: Biallelic mutations in nucleoporin NUP88 cause lethal fetal akinesia deformation sequence
Source: PLoS Genet. 2018 Dec 13;14(12):e1007845. doi: 10.1371/journal.pgen.1007845 (PMC6307818; doi:10.1371/journal.pgen.1007845)
Supplement: S1 Methods — (DOCX) [file pgen.1007845.s001.docx]

**Supplementary Methods**

**Whole-mount in situ hybridization**

The DIG-labeled *nup88* probe DIG was synthesized by antisense T7 RNA polymerase transcription as previously described [1] using the following primers nup88-ISH-Fo: ATTTAGGTGACACTATAGCATCGAGACCCTCTGTGTCA and nup88-ISH-Re: TAATACGACTCACTATAGGGAGCGATGTGCTCTCCTTGTT. At 24 hpf, 0.2 mM 1-phenyl-2-thiourea was added to embryos to block pigment formation. The embryos were fixed at the requisite stage in 4% paraformaldehyde overnight at 4°C and processed for WISH as described previously in [1].

**Whole-mount immunofluorescence**

For cryosections, zebrafish embryos were fixed in 4% PFA for 3 h at RT, washed in PBS and cryoprotected in 30% sucrose in PBS. Samples were embedded in a mix of 7.5% gelantine and 15% sucrose in PBS and frozen for cryostat sections (15 µm thickness). After sectioning, samples were washed 3 times for 5 min in a coplin jar, before incubation in blocking solution (PBS containing 10% FCS, 1% DMSO, 0.3% Triton X-100) for 1 h at RT in a humidification chamber. Primary antibody (mAb414, 1:5000) was incubated overnight at 4 °C. Samples were washed 3 times for 15 min in PBST and secondary anti-mouse Rhodamine-conjugated antibody (1:1000) was incubated for 1 h at RT. Samples were washed 3 times for 15 min in PBST, mounted in mounting medium containing DAPI. Images were recorded at a Zeiss LSM-710 confocal microscope .

**Bacterial expression of recombinant GST-tagged NUP88**

pGEX-cs and pGEX-cs-NUP88 were as previously described [2]. pGEX-cs-NUP88 D434Y was generated by site-directed mutagenesis using listed in Supplementary Table 2.

Recombinant GST-tagged proteins were expressed in *E. coli* BL21-codon plus(DE3)-RIPL strain, as previously described[2]. In brief, BL21 cells were transformed with pGEX-cs, pGEX-cs-Nup88 and pGEX-cs-NUP88 D434Y, plated and grown over night at 37°C. Single colonies were grown in 5 ml LB medium supplemented with ampicillin (Amp) and chloramphenicol (Cam) for 5 to 6 h, transferred into 30 ml LB medium containing Amp/Cam and grown for 16 h at 37°C. 500 ml LB medium containing Amp/Cam were then inoculated to an initial OD_600_ of 0.15 and cultures were grown to OD_600_ of 0.4-0.6 at 37°C. Protein expression was induced by adding 0.5 mM IPTG and the cultures were further grown for 5 h at 30°C or for 16 h at 23°C. Cells were pelleted by centrifugation at 3.220 xg at 4°C for 15 min and stored at -20°C.

For cell lysis, pellets were thawed on ice, resuspended in 7.5 ml of 2x PBS, 1 % Triton X-100, 0.5 mM PMSF supplemented with proteases inhibitors (cOmplete EDTA free; Roche) and lysed by sonication (three cycles of 30 seconds, pulses with 1 min pause in between). Cellular debris were then pelleted by centrifugation at 16.100 xg at 4°C for 10 min, pellets were resuspended in 2x Laemmli buffer, and supernatants were aliquoted and stored at -80°C.

**Preparation of total HeLa protein extracts**

HeLa were grown to confluency, washed once in PBS, harvested using a cell scraper, collected into tubes and pelleted by centrifugation at 290 xg for 7 min at 4°C. Pellets were resuspended in IPH buffer (50 mM Tris-HCl, pH 8, 150 mM NaCl, 5 mM EDTA, 0.5 % IGEPAL CA-630 (v/v; Sigma-Aldrich) supplemented with proteinases inhibitors (cOmplete EDTA free; Roche). The lysate was next incubated for 15 min on ice, thereby briefly vortexed every 5 min, and cleared by centrifugation at 16.100 xg at 4°C. Protein concentration was determined using a Bradford assay.

**GST pull-down assay**

Prewashed glutathione sepharose beads (20 μl) were incubated with 1 ml GST, GST-NUP88 or GST-NUP88 D434Y lysate for 2 h at 4°C, while rotating. After centrifugation at 4°C the supernatant was discarded and the beads were then washed two times with bacteria lysis buffer and twice with binding buffer (0.15 M NaCl, 50 mM HEPES, pH 7.4, 1 mM DTT, 0.1 % IGEPAL CA-630 (v/v; SigmaAldrich) supplemented with proteases inhibitors (cOmplete, EDTA free; Roche). The beads were incubated for 1 hour at 4°C with 1 mg of the total HeLa protein extract in 800 μl binding buffer, while rotating. After centrifugation at 4°C, the supernatant was collected and the beads were washed three times with binding buffer. Bound proteins were eluted using 2x Laemmli buffer, separated by SDS-PAGE, and analysed by immunoblotting.

**Nuclear import and export assays**

For co-expression studies, plasmids coding for wild-type or mutant FLAG-tagged NUP88 were co-transfected with plasmids coding for GFP-tagged reporter proteins (NES-GFP-cNLS, NES-GFP-M9, NES-GFP, GFP-mTor, GFP-SQSTM, GFP-TFEB) using the calcium phosphate method (REF: Ausubel FM, Brent R, Kingston RE, Moore DD, Seidman JG, Smith JA, Struhl K. Current Protocols in Molecular Biology. New York: Greene Publishing Associates and Wiley-Interscience; 1994. ). pEGFP-N1-TFEB was a gift from Shawn Ferguson (Addgene plasmid #38119) [3]. Details about the other reporter plasmids will be provided upon request.

After 24 h, cells were analysed by indirect immunofluorescence, using mouse-anti-FLAG (Sigma, F3165) as primary and donkey-anti-mouse Alexa Fluor 594 (ThermoFisher, A21203) as secondary antibody.

**References**

1. Thisse C, Thisse B. High-resolution in situ hybridization to whole-mount zebrafish embryos. Nat Protoc. 2008;3(1):59-69. Epub 2008/01/15. doi: 10.1038/nprot.2007.514. PubMed PMID: 18193022.

2. Lussi YC, Hugi I, Laurell E, Kutay U, Fahrenkrog B. The nucleoporin Nup88 is interacting with nuclear lamin A. Mol Biol Cell. 2011;22(7):1080-90. doi: 10.1091/mbc.E10-05-0463. PubMed PMID: 21289091; PubMed Central PMCID: PMC3069011.

3. Roczniak-Ferguson A, Petit CS, Froehlich F, Qian S, Ky J, Angarola B, et al. The transcription factor TFEB links mTORC1 signaling to transcriptional control of lysosome homeostasis. Sci Signal. 2012;5(228):ra42. Epub 2012/06/14. doi: 10.1126/scisignal.2002790. PubMed PMID: 22692423; PubMed Central PMCID: PMCPMC3437338.
