# Supplementary figures and images for: Biallelic mutations in nucleoporin NUP88 cause lethal fetal akinesia deformation sequence
Source: PLoS Genet. 2018 Dec 13;14(12):e1007845. doi: 10.1371/journal.pgen.1007845 (PMC6307818; doi:10.1371/journal.pgen.1007845)

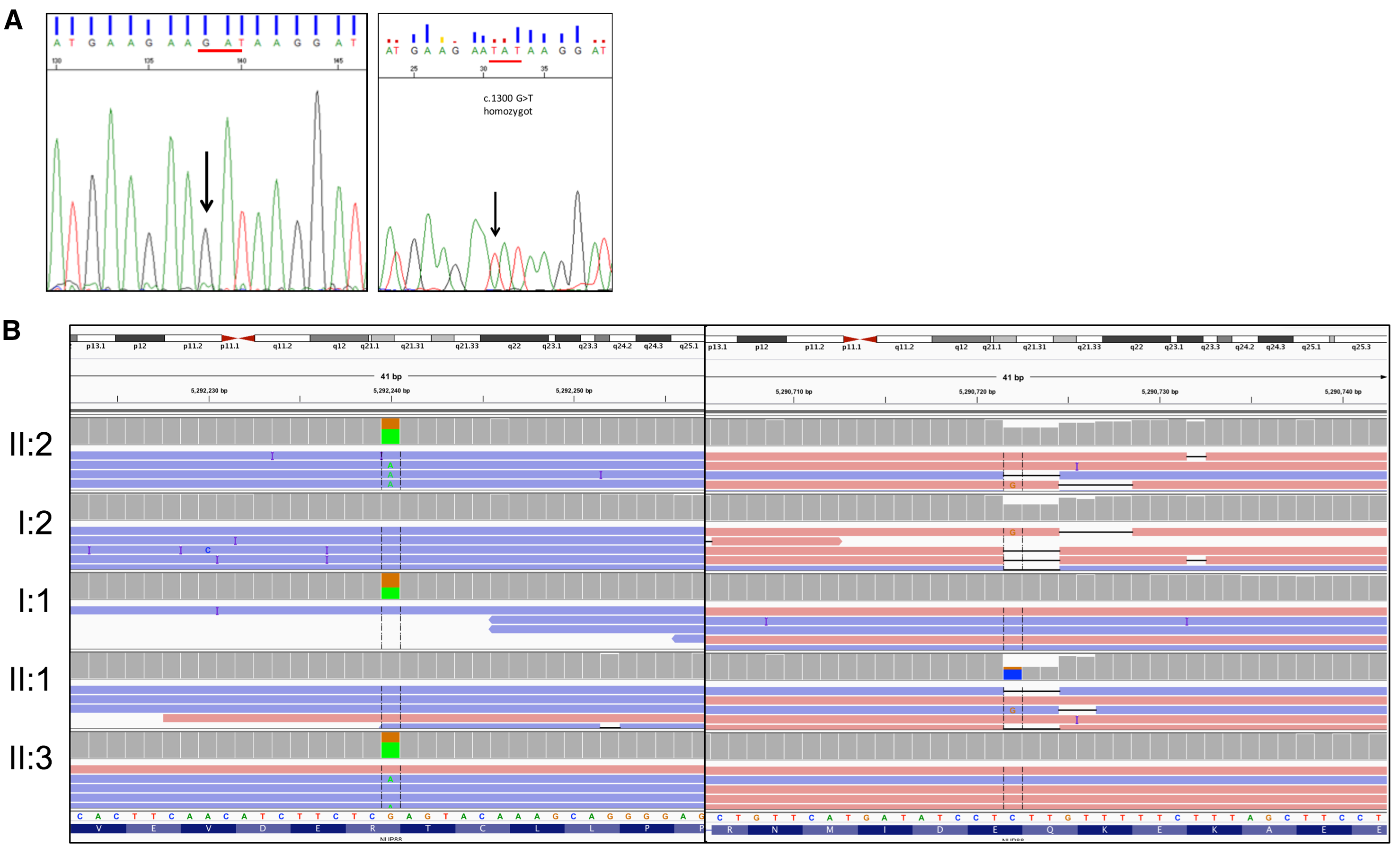

Supplement: S1 Fig — (A) Sanger sequencing chromatograms of control (left) and fetal (fetus 5, Family 1) DNA samples identifying the NM_002532.5 variant c.1300G>T. Exome sequencing (B) of fetus 2, Family 2. (TIFF) [file pgen.1007845.s002.tiff]

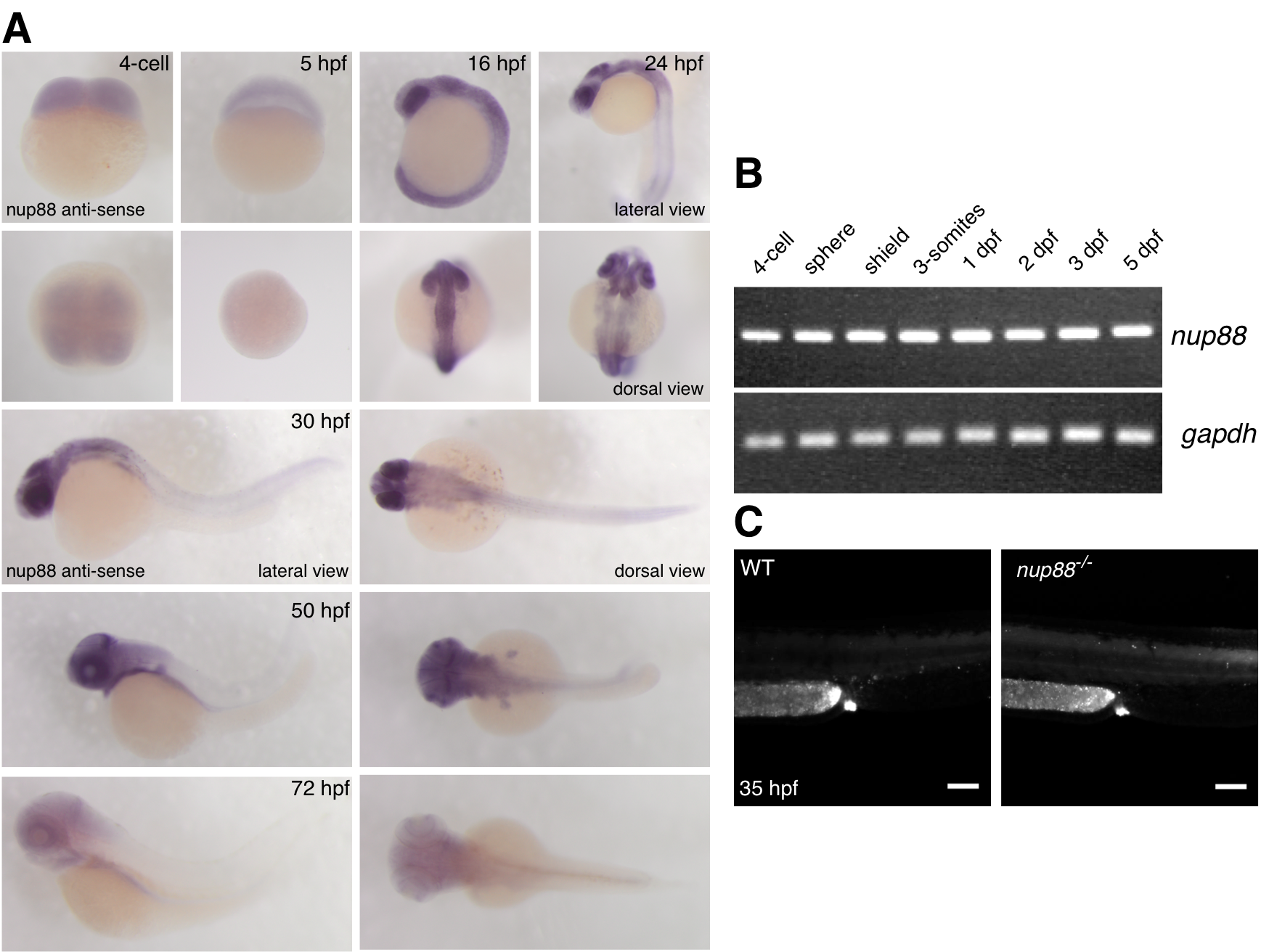

Supplement: S2 Fig — (A) Expression of nup88 in developing zebrafish. Transcripts of nup88 were analyzed by whole-mount in situ hybridization at the indicated developmental stages. nup88 transcripts are maternally deposited during zebrafish oogenesis (4-cell stage). nup88 is further expressed upon zygotic genome activation subsequently to mid-blastula transition (5 hpf and later). nup88 is ubiquitously expressed up to 16 hpf and from 24 hpf prominent in the eye, brain and anterior trunk. nup88 transcripts in somites are low (30 hpf—72 hpf). (B) The presence of maternally deposited nup88 and zygotic nup88 transcripts was confirmed by RT-PCR at stages 4-cell to 5 dpf. actb2, gapdh and ybx1 were used as controls. (C) Acridine orange staining of the tail region of wild-type and nup88-/- mutants at 36 hpf. No major apoptotic events were detected. Shown are confocal images. Scale bars, 100 μm. (TIFF) [file pgen.1007845.s003.tiff]

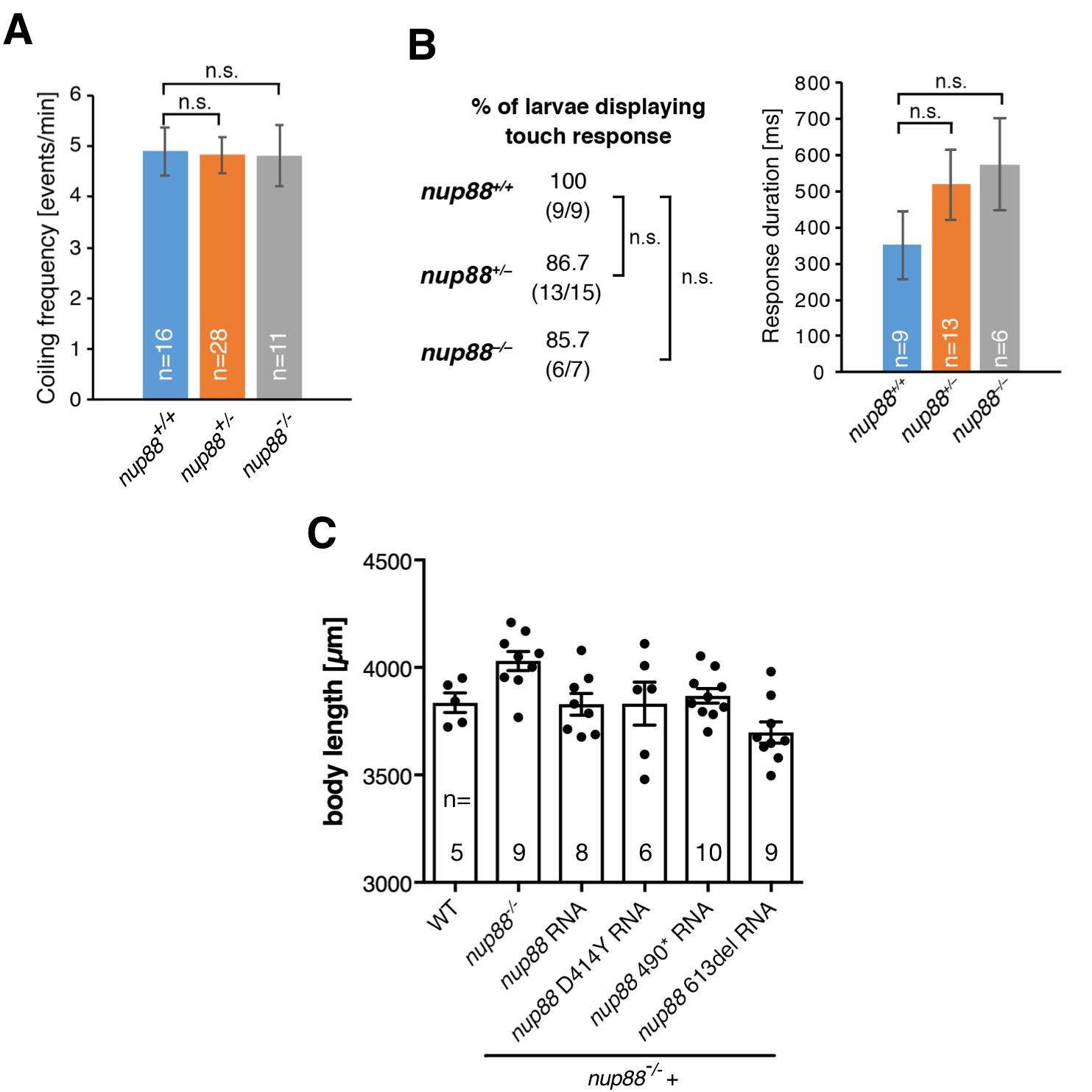

Supplement: S3 Fig — (A) Spontaneous movement (coiling behavior) of 22–24 hpf nup88+/+, nup88+/- and nup88-/- embryos is identical. (B) Touch response is not impaired in nup88 mutant larvae at 3 dpf stage of development. Quantification of percentages of larvae displaying touch-induced escape response (left) and response duration (right) in nup88+/+, nup88+/- and nup88-/- embryos. n.s., not significant, two-tailed t-test (A, B right) or two-tailed Fisher exact test (B left). Data are shown as mean ± SEM. n is number of embryos/larvae analyzed. (C) Quantification of body length after microinjection of wild-type or the respective mutant nup88 were at the one-cell stage. Body length was evaluated at 5 dpf and revealed non-statistically significant differences were detected. Data are shown as mean ± SEM. n is number of embryos/larvae analyzed. (TIFF) [file pgen.1007845.s004.tiff]

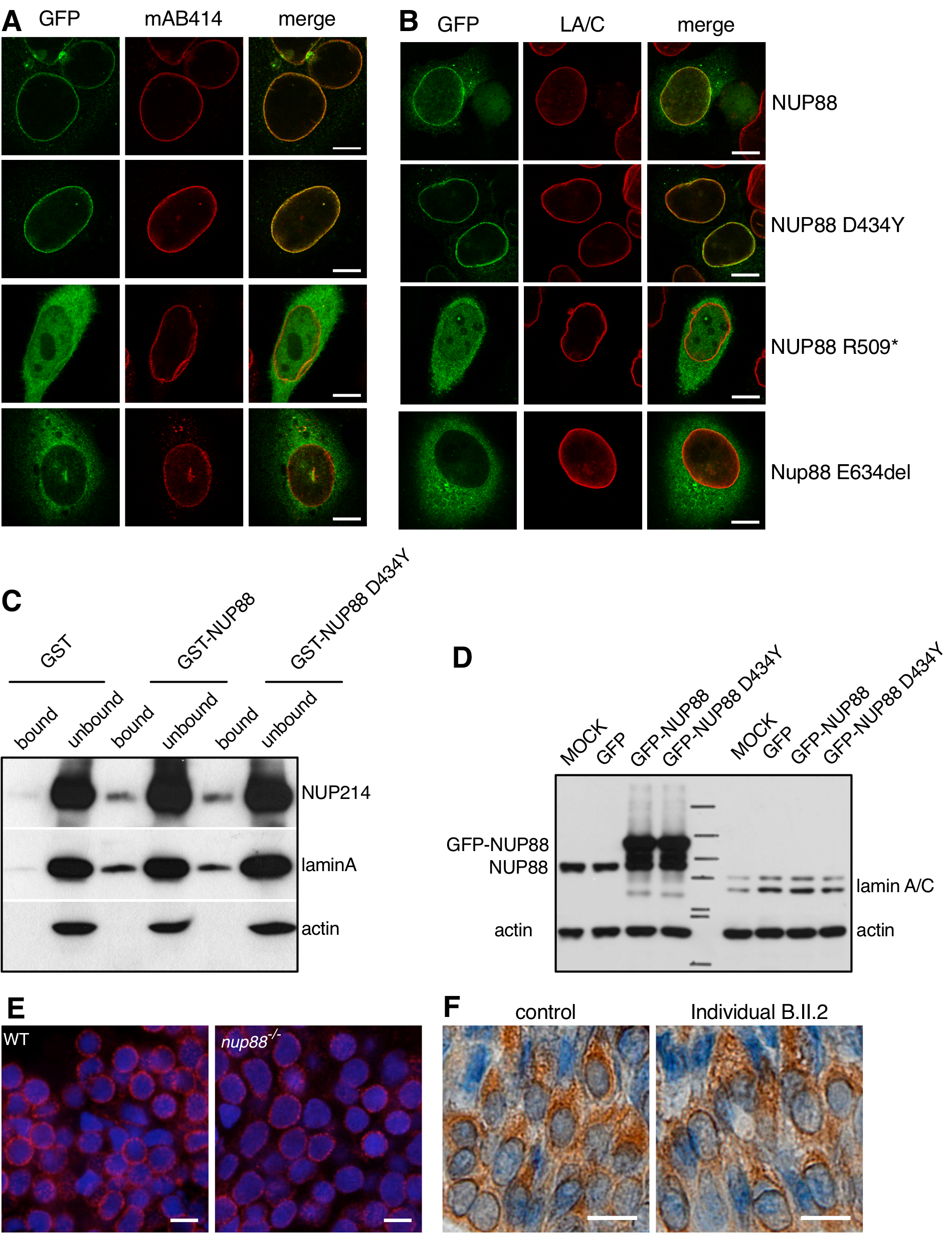

Supplement: S4 Fig — (A) All NUP88 mutants co-localize with the NPC-specific mAB414 antibodies in HeLa cells. Wild-type NUP88, NUP88 D434Y, and NUP88 E634del localize to the NE and the cytoplasm, whereas NUP88 R509* can additionally be found in the nucleus. (B) Nuclear envelope proteins remain unaffected in the presence of mutant NUP88 based on lamin A/C distribution in HeLa cells overexpressing GFP-NUP88 and GFP-NUP88 disease-related mutants. Cells in (A) and (B) were analyzed by indirect immunofluorescence microscopy. Shown are confocal sections on the midplane of the nuclear envelope. Scale bars, 10 μm. (C) Bacterially expressed glutathione-S-transferase (GST), GST-NUP88 and GST-NUP88D434Y were bound to prewashed glutathione sepharose beads and incubated with a total HeLa protein extract. Proteins were eluted using Laemmli buffer and bound and unbound fractions were analyzed by immunoblotting using anti-lamin A, anti-Nup214, and anti-actin antibodies. (D) HeLa cells transiently expressing green-fluorescent protein (GFP), GFP-NUP88 and GFP-NUP88 D434Y were lysed and subjected to Western blot analysis using anti-NUP88, anti-lamin A/C antibodies. Actin served as a loading control. NPCs show normal distribution in (E) the wild-type (WT) and nup88-/- zebrafish as well as in (F) histological muscle sections from individual B.II.2 and a control fetus. Shown are confocal images of sagittal cryo-sections of the diencephalon of 5 dpf zebrafish larvae and bright-field images of paraffin-embedded skeletal muscle section, respectively. NPCs were visualized using the NPC-specific antibody mAB414 (red in (E), brown in (F)). Scale bars: 5 μm (E), 20 μm (F). (TIFF) [file pgen.1007845.s005.tiff]

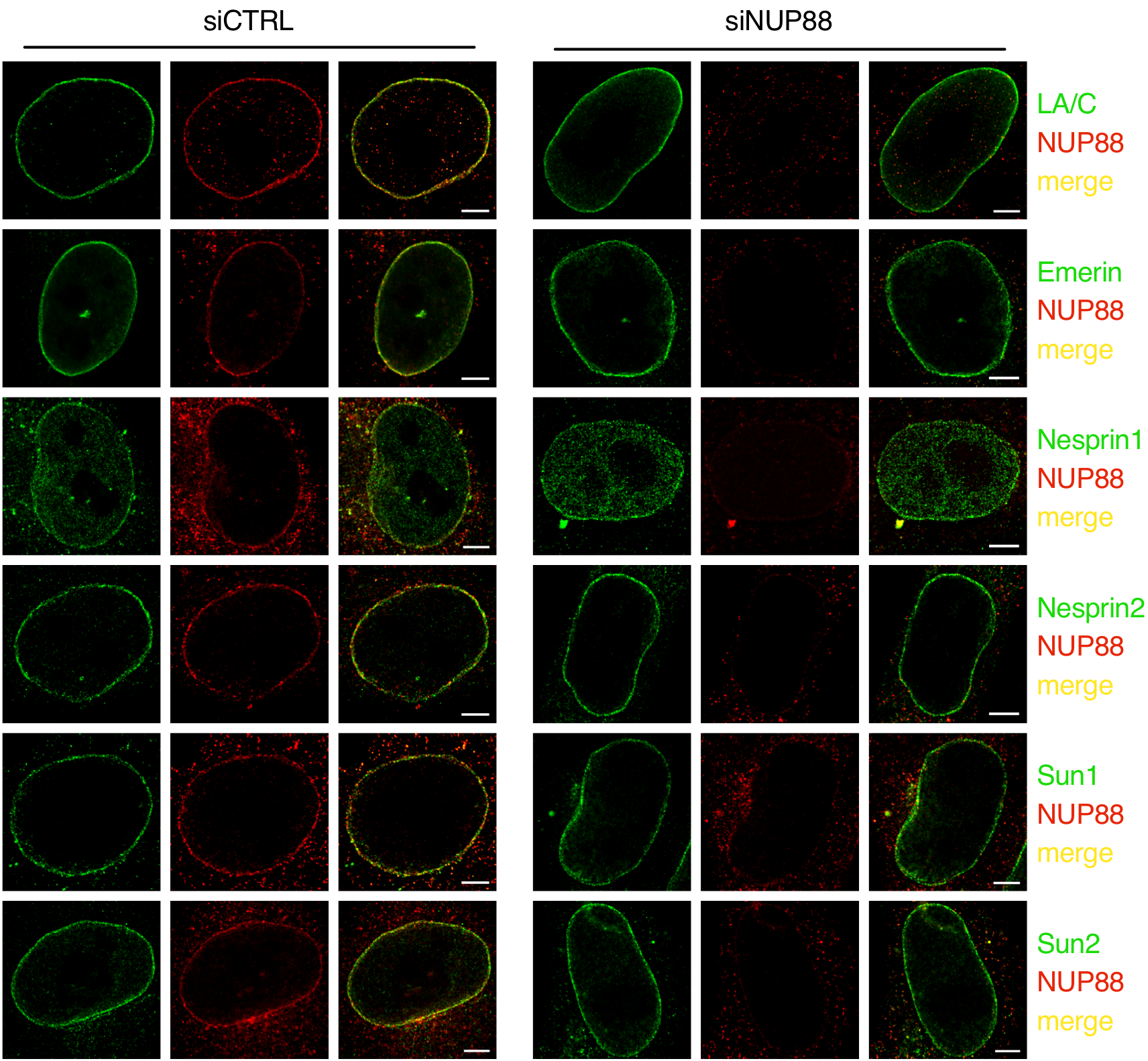

Supplement: S5 Fig — Nuclear envelope proteins remain unaffected in cells depleted for NUP88. Lamin A/C, emerin, Nesprin 1, Nesprin 2, Sun1 and Sun2 distribution are similar in HeLa cells treated with control siRNA and siRNA against NUP88, respectively. Cells were analyzed by indirect immunofluorescence microscopy. Shown are confocal sections on the midplane of the nuclear envelope. Scale bars, 5 μm. (TIFF) [file pgen.1007845.s006.tiff]

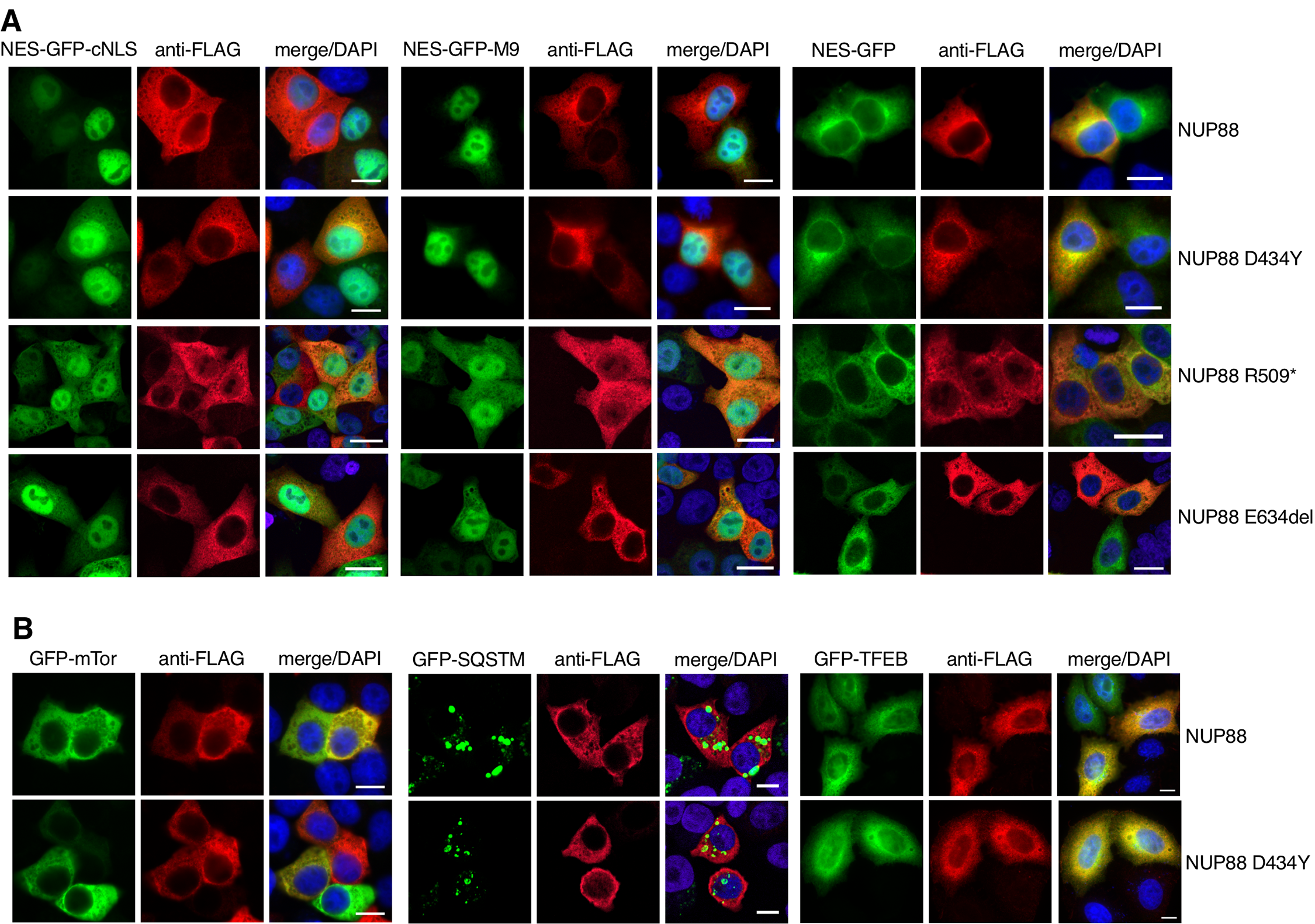

Supplement: S6 Fig — Cells were transfected with plasmids coding for wild-type or mutant FLAG-tagged NUP88 and for the nucleocytoplasmic transport substrates NES-GFP-cNLS, NES-GFP-M9 and GFP-NES (A) or the CRM1-cargoes GFP-mTor, GFP-SQSTM and GFP-TFEB (B). After 24 h, cells were subjected to indirect immunofluorescence and analyzed by confocal microscopy. (TIFF) [file pgen.1007845.s007.tiff]
