# Supplementary material for: Biallelic mutations in nucleoporin NUP88 cause lethal fetal akinesia deformation sequence
Source: PLoS Genet. 2018 Dec 13;14(12):e1007845. doi: 10.1371/journal.pgen.1007845 (PMC6307818; doi:10.1371/journal.pgen.1007845)
Supplement: S1 Table — (DOCX) [file pgen.1007845.s008.docx]

**Supplementary Table 1**: Prediction tools

| **Mutation**  **Protein** | **c.1300C>T**  **p.D434Y** | **c.1525C>T**  **p.R509*** | **c.1899_1901del**  **p.E634del** |
| --- | --- | --- | --- |
| SIFT prediction  Provean score | Deleterious  -3.768 (threshold: -2.5) | Deleterious  NA | Deleterious  -9.595 (threshold: -2.5) |
| Polyphen-2 prediction  Polyphen-2 score | Probably damaging  0.993 (sensitivity: 0.47; specificity:0.96) | Probably damaging | Probably damaging |
| Mutation Taster prediction  Mutation Taster probability  Mutation Taster predicted change | Disease causing  0.99999984445111  amino acid sequence changed; protein features (might be) affected; splice site changes | Disease causing  1  NMD; amino acid sequence changed; protein features (might be) affected; splice site changes | Disease causing  0.999999975957048  amino acid sequence changed; protein features (might be) affected; splice site changes |
