# Supplementary material for: Biallelic mutations in nucleoporin NUP88 cause lethal fetal akinesia deformation sequence
Source: PLoS Genet. 2018 Dec 13;14(12):e1007845. doi: 10.1371/journal.pgen.1007845 (PMC6307818; doi:10.1371/journal.pgen.1007845)
Supplement: S2 Table — (DOCX) [file pgen.1007845.s009.docx]

**Supplementary Table 2**

Sequences of oligonucleotide primers used for PCR and Sanger DNA sequencing

| Name | Description | Sequence (5’-3’) |
| --- | --- | --- |
| nup88-geno-Fo | Genotyping PCR of *D. rerio* *nup88* | GTTCTTATGCGGCCTCTTTG |
| nup88-geno-Rev | Genotyping PCR of *D. rerio* *nup88* | TCAGAAAGGTCTCTCCATTGC |
| nup88-For | Infusion cloning PCR *D. rerio* *nup88* | CTTGTTCTTTTTGCAGGATCCGCCACCATGGCGTCGCTTGCGGG |
| nup88-Rev | Infusion cloning PCR *D rerio nup88* | CTATAGTTCTAGAGGCTCGAGTCAAGAGCACAGCAGCGGAGGAGAAG |
| nup88 c.1240G>T-FOR | Site-directed mutagenesis of *D. rerio* *nup88* | CTGCAGACTGTCTTTATACTCCTCATCAGACTCCA |
| nup88 c.1240G>T-REV | Site-directed mutagenesis of *D. rerio* *nup88* | TGGAGTCTGATGAGGAGTATAAAGACAGTCTGCAG |
| nup88 c.1468-1470>TGA-FOR | Site-directed mutagenesis of *D. rerio* *nup88* | GCTGCTGTGCTCTTAACATCCAGGGGCGG |
| nup88 c.1468-1470>TGA-REV | Site-directed mutagenesis of *D. rerio* *nup88* | CCGCCCCTGGATGTTAAGAGCACAGCAGC |
| nup88 c.1837_1839del-FOR | Site-directed mutagenesis of *D. rerio* *nup88* | GAAGATGCCAAATCTCGTCAAGCCATCATGAACAGAG |
| nup88 c.1837_1839del-REV | Site-directed mutagenesis of *D. rerio* *nup88* | CTCTGTTCATGATGGCTTGACGATATTTGGCATCTTC |
| nup88-ISH-FOR | Whole-mount in situ hybridisation of *D. rerio* *nup88* | ATTTAGGTGACACTATAGCATCGAGACCCTCTGTGTCA |
| nup88-ISH-REV | Whole-mount in situ hybridisation of *D. rerio* *nup88* | TAATACGACTCACTATAGGGAGCGATGTGCTCTCCTTGTT |
| gapdh-FOR | qRT-PCR *D. rerio* | CAGTCGCTGATGTGTCCGTTGTC |
| gapdh-REV | qRT-PCR *D. rerio* | GAGACGAACGCAGCGTCTCGTAG |
| actb2-FOR | qRT-PCR *D. rerio* | GCAGAAGGAGATCACATCCCTGGC |
| actb2-REV | qRT-PCR *D. rerio* | CATTGCCGTCACCTTCACCGTTC |
| ybx1-FOR | qRT-PCR *D. rerio* | GTAGCGTTGGGGACGGAGAGACTG |
| ybx1-REV | qRT-PCR *D. rerio* | GGCCGGATGGTCTGGATGTTTAAG |
| nup88-FOR | qRT-PCR *D. rerio* | GTTCTTATGCGGCCTCTTTG |
| nup88-REV | qRT-PCR *D. rerio* | CATCGGCTTCCCTAAACTACC |
| zf-rapsn FOR1 | qPCR *D. rerio* | CAGGCATTGTGTCTGCTCAA |
| zf-rapsn REV1 | qPCR *D. rerio* | CTGCAGAATCCAGCACTTCC |
| hNup88 D434Y-Sense | Site-directed mutagenesis of human *NUP88* | GAGTTCCTGTAAACTTATCCTTATATTCTTCATCTGATCCAAGAAATT |
| hNup88 D434Y-Antisense | Site-directed mutagenesis of human *NUP88* | AATTTCTTGGATCAGATGAAGAATATAAGGATAGTTTACAGGAACTC |
| hNup88 R509 Stop-Sense | Site-directed mutagenesis of human *NUP88* | CTCCCCTGCTTTGTACTTGAGAAGATGTTGAAGTG |
| hNup88 R509 Stop-Antisense | Site-directed mutagenesis of human *NUP88* | CACTTCAACATCTTCTCAAGTACAAAGCAGGGGAG |
| hNup88 E634del-Sense | Site-directed mutagenesis of human *NUP88* | ctgacaaatatgaggaagctaaagaaaaacaggatatcatgaacagg |
| hNup88 E634del-Antisense | Site-directed mutagenesis of human *NUP88* | cctgttcatgatatcctgtttttctttagcttcctcatatttgtcag |
